# Bladder wrap: a technique to restore continence in an incompetent vesicocutaneous diversion

**DOI:** 10.1308/003588412X13373405386015j

**Published:** 2012-09

**Authors:** A Mangera, I Edhem

**Affiliations:** Rotherham NHS Foundation Trust,UK

## BACKGROUND

Paul Mitrofanoff popularised the flap valve technique for creating continent urinary diversions.^1^ The eponymous technique allows the use of several tissues to create a catheterisable vesicocutaneous diversion including the vermiform appendix, small/large bowel and fallopian tubes. Three main techniques are used to maintain continence of the stoma. These involve tunnelling of the proximal portion of the diversion into the bladder to form a flap, nipple or hydraulic valve, with the flap being the most common.^2^ Urinary leak occurs in approximately 4% of patients after the flap procedure and is independent of the tissue used.^3^ These patients often require revision of the diversion or creation of a new stoma altogether.^4^ Here we describe a less radical technique.

## TECHNIQUE

**Figure 1:**
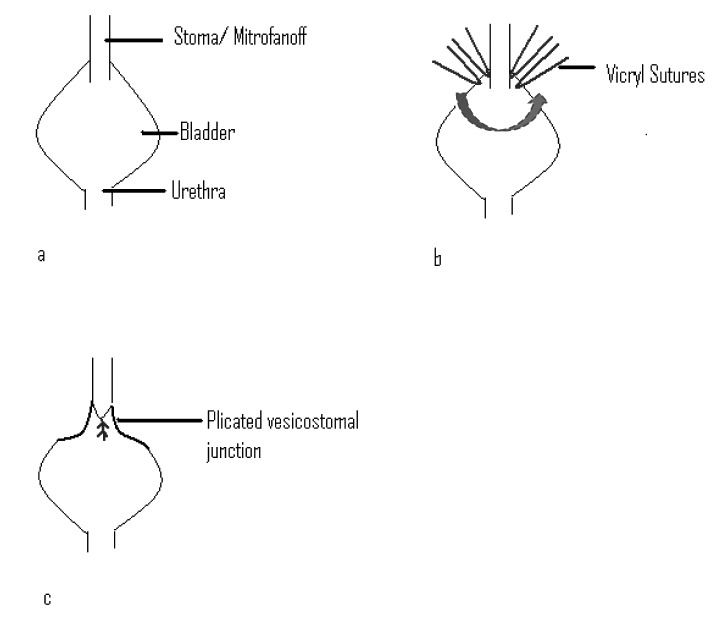
Formation of a bladder wrap: a) existing anatomy; b) sutures are placed at the vesicostomal junction into the detrusor; c) sutures are tied, plicating the junction.

After dissection through the abdominal wall, the anastomosis between the bladder and stoma is identified. The lateral edges of the bladder are plicated around the anastomosis in a manner similar to that of a Nissen fundoplication using 3/0 Vicryl® sutures (Ethicon Inc, Somerville, NJ, US) (Fig 1). The surgeon must leave enough slack on the suture to ensure easy passage of a 12Fr LoFric® catheter (Astra Tech, Stonehouse, UK) into the bladder. Having had this procedure, three patients regained continence with a maximum follow-up of 16 months.

## DISCUSSION

The technique achieves continence by acting as a compression valve around the proximal anastomosis as the bladder fills. This technique is described in the literature only in the prevention of vesicoureteric reflux in animals.^5^ We advocate its use as first line management in patients with an incontinent vesicocutaneous diversion.
